# Electrically Tunable Polymer Whispering-Gallery-Mode Laser

**DOI:** 10.3390/ma15144812

**Published:** 2022-07-10

**Authors:** Fangyuan Liu, Junhua Tong, Zhiyang Xu, Kun Ge, Jun Ruan, Libin Cui, Tianrui Zhai

**Affiliations:** 1 College of Physics and Optoelectronics, Faculty of Science, Beijing University of Technology, Beijing 100124, China; fyliu@emails.bjut.edu.cn (F.L.); xu.zhiyang@hotmail.com (Z.X.); gekun@emails.bjut.edu.cn (K.G.); ruanj@emails.bjut.edu.cn (J.R.); 2 College of Mathematics and Physics, Beijing University of Chemical Technology, Beijing 100029, China; tongjh@buct.edu.cn

**Keywords:** microring cavity, PFO, PMN-PT piezoelectric crystal, WGM, electrically tunable laser

## Abstract

Microlasers hold great promise for the development of photonics and optoelectronics. At present, tunable microcavity lasers, especially regarding in situ dynamic tuning, are still the focus of research. In this study, we combined a 0.7Pb(Mg_1/3_Nb_2/3_)O_3_-0.3PbTiO_3_ (PMN-PT) piezoelectric crystal with a Poly [9,9-dioctylfluorenyl-2,7-diyl] (PFO) microring cavity to realize a high-quality, electrically tunable, whispering-gallery-mode (WGM) laser. The dependence of the laser properties on the diameter of the microrings, including the laser spectrum and quality (*Q*) value, was investigated. It was found that with an increase in microring diameter, the laser emission redshifted, and the *Q* value increased. In addition, the device effectively achieved a blueshift under an applied electric field, and the wavelength tuning range was 0.71 nm. This work provides a method for in situ dynamic spectral modulation of microcavity lasers, and is expected to provide inspiration for the application of integrated photonics technology.

## 1. Introduction

In recent years, microcavity-based lasers have shown great potential in applications such as optical integration [1,2], on-chip optical communication [3,4], and ultrasensitive sensing [5,6,7]. Among all configurations, whispering-gallery-mode (WGM) microcavities have been intensively investigated due to their excellent optical properties. Continuous total internal reflection of light on smooth surfaces of WGM microcavities, such as microspheres [8,9], microdisks [10,11], microrings [12,13], microbottles [14,15], microbubbles [16,17], and microfibers [18], leads to repeated self-enhancement of light. Accordingly, WGM lasers always possess a high quality (*Q*) factor, small mode volume, and low threshold. At present, various shapes of WGM microcavities have been successfully prepared by electron beam lithography [19], chemical synthesis [20], electrostatic spinning [21], inkjet printing [22], and other methods. Among them, the inkjet printing method can produce a high-quality WGM microcavity structure quickly and with low cost and high precision.

Wavelength-tunable microlasers, as indispensable components in various photonic devices, have attracted great interest. The wavelength variability of microcavity lasers provides the possibility of integrating photonic devices with more functions and is a key requirement for the generation of more compact devices. In previous studies, researchers have achieved wide-range laser wavelength tuning in discrete microcavities by controlling the size of the microcavities [23,24] and the synthesis of gain materials [25,26]. Heterocoupled microresonators composed of distinct cavities are a novel approach for generating tunable multicolor single-mode lasers [27,28,29]. Sun’s group [30,31] reported a series of mechanical bending tuning of WGM lasers integrated on flexible substrates. Zhao’s group [32,33] constructed broadband tunable microlasers based on the controlled ICT process for a specific gain material. In other work, researchers have also altered the ambient temperature [29] and medium [34] to affect the laser emission. In addition, some researchers have shown that electronically tunable distributed feedback (DFB) lasers can be achieved through electroactive dielectric elastomer actuators [35] and III–V InGaAsP tuning layers [36]. To date, there have been a few studies on WGM electrical tuning; microstructural fibers based on dual-frequency liquid crystal (DFLCs) [37] and metal-dielectric core–shell hybrid microcavities with thermo-optical effects [38] provide WGM tuning schemes for wavelength shifting by applied electric fields.

Here, we propose an electrotunable microlaser to tune the emission wavelength in situ through piezoelectric effect-induced strain. Microring resonators were prepared by the inkjet printing method, which has a low cost and is of high quality and can be prepared in batches. Wavelength tuning was achieved by fabricating microring resonators on 0.7Pb(Mg_1/3_Nb_2/3_)O_3_ -0.3PbTiO_3_ (PMN-PT) piezoelectric single crystals with ultrahigh piezoelectric strain constants, d_31_ up to ~−3000 pm/V, which provide strain. Compared to using static strain, the unique feature of using piezoelectric strain is that piezoelectric strain can provide continuous dynamic modulation under an external electric field. This approach is suitable for a variety of gain materials and microcavities. In addition, this modulation is extremely sensitive due to the rapid response of PMN-PT piezoelectric crystals to electric fields.

## 2. Fabrication and Measurement

**Figure 1:**
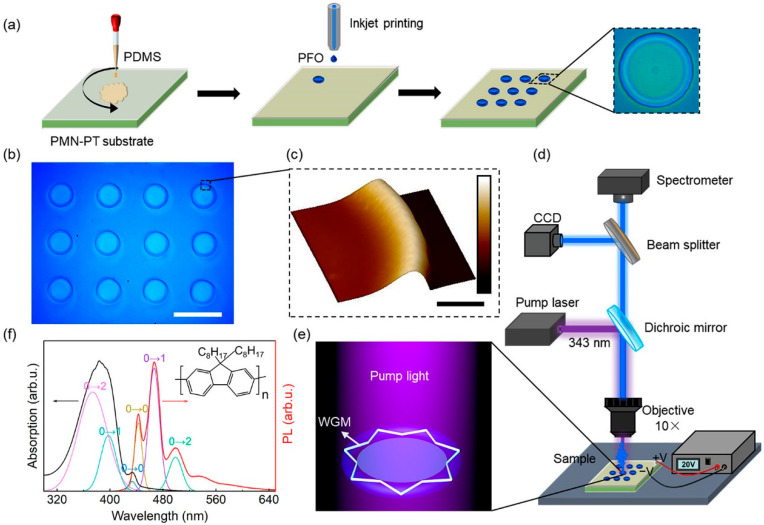
Fabrication and measurement of the PFO microring laser. (**a**) Schematic illustration of the fabrication progress of the microring resonator. (**b**) Microscope image showing the printed microring array. The scale bar is 100 μm. (**c**) AFM image of the microring structure. The scale bar is 10 μm. (**d**) Experimental setup for excitation and signal collection of microring resonators. (**e**) Schematic diagram of feedback light propagation in a microring cavity. (**f**) The normalized absorption (black line) and emission (red line) spectra of PFO.

The fabrication of a microring laser is illustrated in Figure 1a. The device was fabricated by the inkjet printing method, and the specific process is shown in Figure 1a. A 30 nm gold electrode was deposited on the upper and bottom surfaces of the PMN-PT piezoelectric single crystal by magnetron sputtering (CK-450, Baijujie Scientific Instrument Co., Ltd., Shenyang, China) before the microcavity was prepared, and the electrode was annealed at 400 °C for 110 min to make it adhere firmly to the substrate. Then, PDMS was rotated on the substrate at 3000 rpm to provide a hydrophobic environment for the subsequent preparation of microcavities. Poly[9,9-dioctylfluorenyl-2,7-diyl] (PFO) was completely dis-solved in xylene solution at a concentration of 18 mg/mL as the printing ink. A high-precision printer (Microfab JETLAB 4, Microfab Technologies Inc., Shanghai, China) equipped with a 60 μm diameter piezo-driven inkjet nozzle squirted droplets onto the PDMS film to form the microstructure. By setting appropriate operating parameters in Jetlab program, the nozzle was controlled to realize various printing tasks. This method can realize batch preparation of microcavities, and a microscopic (OLS4100, Olympus, Tokyo, Japan) image of the printed microring array is shown in Figure 1b. The jetted PFO solution droplets partially dis-solved the PDMS film and formed a higher ring-shaped structure at the boundary of the droplets on the substrate due to the coffee-ring effect [39,40], as shown in the image in Figure 1c, which was obtained by Atomic Force Microscopy (AFM, Bruker MultiMode 8, Billerica, MA, USA). The microring structure is very smooth, contributing to low loss and a high *Q* factor. Moreover, its diameter could be adjusted by changing the volume of the xylene droplet, as controlled by a piezoelectric-driven inkjet nozzle. In addition, by changing the printing parameters to control the droplet volume, microrings of different sizes can be obtained.

**Figure 2:**
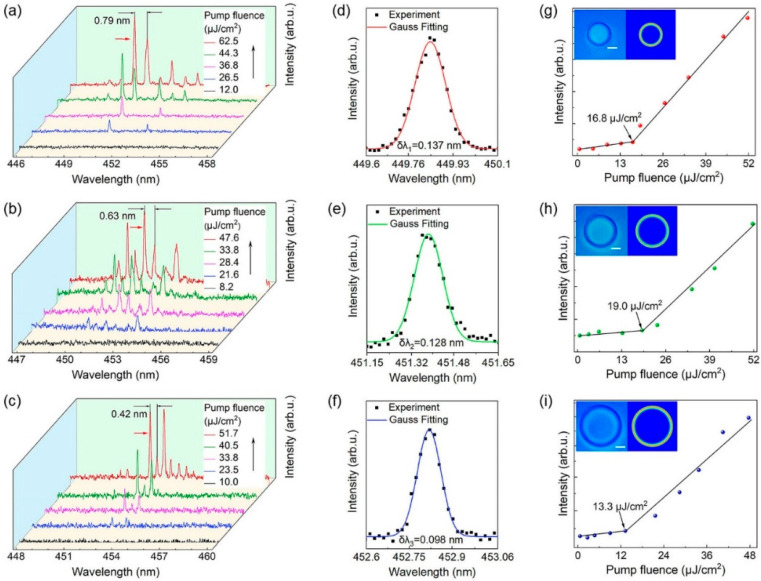
Laser characteristics of the PFO microring lasers. (**a**–**c**) The normalized photoluminescence spectra of microrings at different pump fluences, and the diameters are 53, 67, and 85 μm, respectively. (**d**–**f**) Gaussian fitting of the lasing oscillation mode. (**g**–**i**) Plots of the PL peak intensities versus pump fluence for microrings with diameters of 53, 67, and 85 μm, respectively. Left inset: microscope image of the microrings. The scale bar is 20 μm. Right inset: simulated electric field intensity distribution of the microring resonators.

In the experiment, a microphotoluminescence system, as shown in Figure 1d, was employed to obtain the spectral signals, and a pulsed laser (343 nm wavelength, pulse duration of 10 ns, and repetition rate of 80 Hz) was used as the excitation source for pumping the microring resonator at room temperature. Figure 1e shows a schematic diagram of feedback light propagation in a microring cavity under pumping conditions. The total internal reflection of light occurring in the inner wall of the microring was confined in the microcavity, and the WGM mode was formed after several self-interference intensifications. The gain material used in the experiment was the blue polymer PFO, and its absorption and fluorescence spectra are shown in Figure 1f. PFO is a polyfluorene material with a number average molecular weight (Mn) of 6.9 × 10^4^ and polydispersity (PDI) of 1.33 [41]. It is worth noting that PFO has different morphologies, such as an amorphous phase with an average torsional angle (φ) of main chain 135°, and a so-called β- phase with a relatively flat main chain structure of 160°. The planar configuration results in extended mean conjugation length and more effective electronic delocalization [42]. PFO dis-solved in xylene will produce β-PFO; it has been proven that the PL quantum yield of β-PFO film is significantly higher than that of the amorphous PFO film [41]. The three vibronic bands at 442, 467, and 497 nm in the spontaneous emission spectrum of β-PFO correspond to 0–0, 0–1, and 0–2 transitions, respectively (Figure 2f), which is ascribed to the efficient energy transition from the amorphous section to the β-phase region in the PFO [41]. Relatively low-power-driven PFO microring lasing was realized based on the apparent 0–1 band stimulated transition.

## 3. Results and Discussions

### 3.1. Emission Spectra

The lasing operation of individual microring lasers of various sizes is demonstrated by the spectral analysis shown in Figure 2. Figure 2a–c show the power-dependent lasing spectra of an individual microring pumped at 343 nm, with diameters of 53, 67, and 85 μm, respectively. The corresponding microscopic image and size of the microring are shown in the first illustration in the upper left corner of Figure 2g–i. The focused pulsed laser locally excites the microrings in air, and only a weak spontaneous emission is observed at a reduced pump power density. When the pump intensity increases to the stimulated emission threshold, the enhanced whispering-gallery modes in the microring wall are favorable for light feedback, and the spontaneous emission is transformed into a narrow linewidth laser emission with a strong peak value. Comparing the laser spectra of microcavities of different sizes, a redshift is found with increasing size. According to the resonance conditions of the WGM cavity [43]:

(1) $${m\lambda =Ln}_{eff}$$

where m is the angular mode number, λ is the wavelength of the light in vacuum, $n_{eff}$ is the effective refractive index, and L is the circumference of the microcavity. Clearly, with increasing microcavity size, the wavelength also increases, which fits well with the redshift phenomenon in the experiment.

In addition to the strong amplification of the modal peaks, another essential point is that multiple sharp laser peaks with regular intervals are observed as the pump fluence is increased further. The free spectral range (FSR) is usually defined as the distance between two adjacent angular mode wavelengths, which is a typical size-dependent feature of WGM microcavity resonators. The theoretical calculation of the FSR can be calculated as follows [43]:

(2) $$\mathrm{FSR}=\frac{\lambda^{2}}{{\pi \mathrm{Dn}}_{\mathrm{eff}}}$$

where λ is the peak wavelength, $n_{eff}$ is the effective refractive index, and D is the diameter of the PFO microring obtained under the microscope. From Equation (2), the diameter of the microring cavity is inversely proportional to the FSR. As seen from the PL spectrum in Figure 2, the laser modes become dense as the diameter of the microring increases, which confirms this theory.

The formula for calculating the quality factor is *Q* = *λ*/*δλ*, where *δλ* is the linewidth. Figure 2d–f is the Gaussian function fitting corresponding to the laser peaks marked by the red arrows in Figure 2a–c, respectively, and the *δλ* of the laser peaks is obtained. The *Q* values of the microring lasers with diameters of 53, 67, and 85 μm are calculated to be 3280, 3530, and 4620, respectively. The relation *Q* = 2π*nL*/*λ*(1 − *R*) [44] actively demonstrates that the *Q* value increases with increasing microcavity size, which is due to the increase in cavity length and microcavity-air interface reflectance.

Figure 2g–i plots the PL intensity as a function of pump fluence, corresponding to the three sizes of microrings in Figure 2a–c on the left, respectively. It can be seen from the figure that the lasing peak emission intensity increases dramatically with the excitation power, and the change is nonlinear. For the 53 μm, 67 μm, and 85 μm microrings, the data line turning point indicates the laser thresholds, which are 16.8 μJ/cm^2^, 19.0 μJ/cm^2^, and 13.3 μJ/cm^2^, respectively. The clear threshold behaviour confirms the laser action of the microring lasers. COMSOL software was employed to simulate the WGM mode in the PFO microring resonant cavity, and the simulation parameters, including the geometric radius and effective refractive index, were obtained from the experiment. The electric field intensity distribution in the cross section of the WGM laser is shown in the second picture in the upper left corner of Figure 2g–i. Due to the refractive index difference between the polymer microcavity and the ambient medium, the light is captured in the inner wall of the smooth microcavity by multiple total internal reflection. When the optical path is an integer multiple of wavelength, a stable standing wave is formed in the microcavity. Therefore, a strong local field of laser mode can be observed in the microcavity [41].

### 3.2. Wavelength Tuning

**Figure 3:**
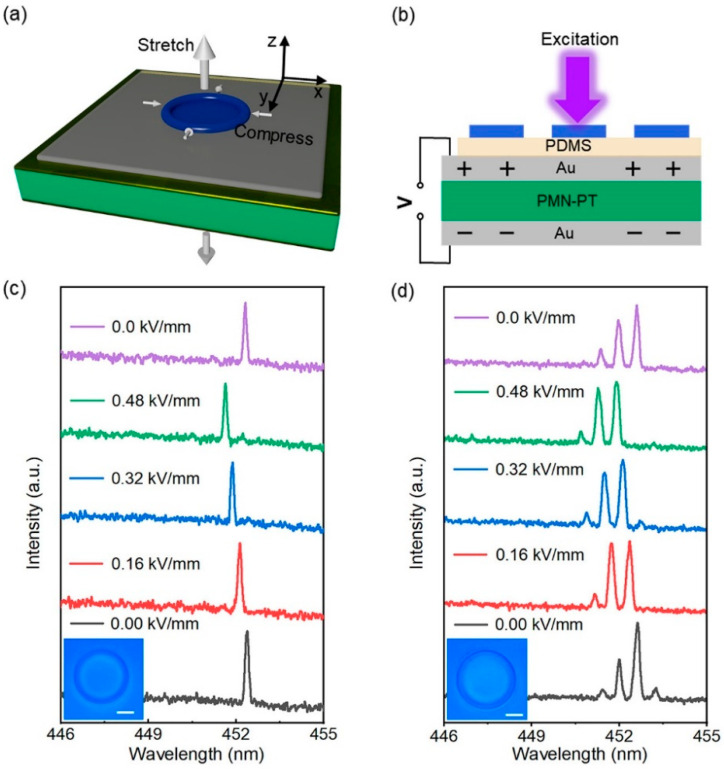
Wavelength tuning of the microring laser. (**a**) Schematic diagram of laser device deformation. (**b**) In situ measurement of laser spectra under an electric field. (**c**) Laser spectra of 58 μm diameter microring under different electric fields. Inset: Microscope image of the microring. The scale bar is 20 μm. (**d**) Laser spectra of 70 μm diameter microring under different electric fields. Inset: microscope image of the microring. The scale bar is 20 μm.

**Figure 4:**
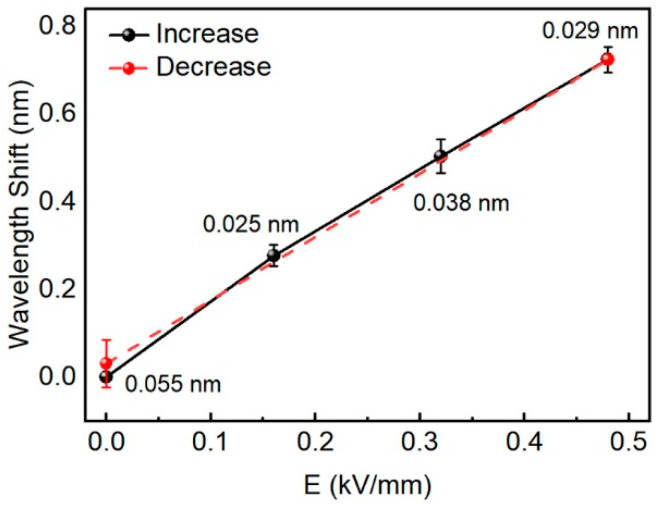
Wavelength tuning of the microring lasers under different electric fields.

By applying an in situ electric field to the PMN-PT piezoelectric single crystal substrate, tuning of the emission wavelength of the microring laser was realized. Prior to the wavelength tuning test, the PMN-PT substrate was polarized at a direct current (DC) field of 0.5 kV/mm. The electric field applied in subsequent electrical tuning should be lower than the intensity of the polarization electric field to obtain a stable electrostrictive effect of PMN-PT piezoelectric substrate. After polarization treatment, the PMN-PT substrate was subjected to an electric field in the same direction as the poling electric field so that the substrate produced a regular strain. When the external electric field was applied to the [110]-oriented PMN-PT substrate along the [110] direction (the *z*-axis in Figure 3a), the transverse strain can be obtained from the piezoelectric equation [45]:

(3) $$S_{1}=s_{11}^{E}T_{1}+d_{31}E_{3}$$

where $s_{11}^{E}$ is the elastic compliance coefficient and $d_{31}$ is the piezoelectric strain constant. Therefore, a positive electric field applied to the *z*-axis direction will induce the shrinkage of the *x*-axis length direction. The device deformation process is shown in Figure 3a. When the applied electric field is in the same direction as the polarization field, the crystal shrinks in the xy-plane. Therefore, the size of the microring cavity is reduced. Compared with other electrostrictive materials, the $d_{31}$ parameter ($d_{31}$ up to ~3000 pm/V) determines the larger transverse deformation of the [110]-oriented PMN-PT crystal under the same electric field. In Reference [43], a large negative linear transverse strain of 0.15% is observed under an electric field of 0.5 kV/mm (normalized strain reaches 3000 pm/V) with minimal hysteresis [45]. Due to the loss caused by the PDMS layer and laser material, the strain transmitted to the microcavity is reduced. However, even small deformations can still significantly affect the behavior of light. Next, an external electric field was applied to the PMN-PT single crystal substrate between the upper and bottom Au electrodes and the laser spectra were simultaneously recorded in situ, as shown in Figure 3b. Figure 3c,d present the normalized laser spectra of PFO microrings, and the insets are the microscope images of the test microrings, with diameters of 58 and 70 μm, respectively. In Figure 3c, the microring laser was excited at a lower pump energy of 24 μJ/cm^2^ and only one mode was observed, and the other microring laser was pumped at 35 μJ/cm^2^. A clear, blue-shifted laser peak can be observed when the electric field varies from 0 to 0.48 kV/mm. Under the action of the electric field, the diameter of the microrings decreases due to the electrostriction of the piezoelectric substrate in the xy-plane, resulting in a blue-shift of the laser peak. Under the electric field of 0.48 kV/mm, the maximum blueshift of the first microring laser reaches 0.73 nm, which is in consistency with the second microring laser (0.71 nm). Although the tuning range of this in situ tunable microring laser is limited compared to some tunable WGM lasers with special gain materials [26,32,33,37] and mechanically tuned WGM lasers in three-dimensional space [30,31], it is ten times that of other WGM lasers tuned in two-dimensional space [29,34,37]. Wavelength tuning at different electric field amplitudes is clearly shown in Figure 4. The red dots correspond to the wavelength shift distance measured for a series of increasing actuation voltages, while the black dots correspond to the return curve. The peak position is almost restored to the initial position after removing the electric field. Under the control of the electric field, the continuous modulation of the laser wavelength is effectively realized.

## 4. Conclusions

In summary, a wavelength-tunable PFO microring WGM laser device is demonstrated utilizing PMN-PT piezoelectric crystals. PFO microring lasers with different diameters were prepared by the inkjet printing method, which has the advantages of low cost, flexible preparation, and batch preparation. The laser spectra of microrings with different diameters are compared, and when the diameter of the microring is 85 μm, the threshold is as low as 13.3 μJ/cm^2^, and the *Q* value reaches 4620. The electrostrain-induced properties of PMN-PT piezoelectric substrates change the laser emission wavelength, which is unique in that it can flexibly realize in situ continuous dynamic tuning in two-dimensional space. Wavelength tuning of about 0.7 nm can be achieved by applying a DC electric field of 0.48 kV/mm. This tuning method has strong repeatability and can accurately control wavelength shift under different electric field intensities. Furthermore, on the basis of this method, microcavities with different geometric shapes or coupled microcavities can be prepared to realize wavelength tuning of a single longitudinal mode laser. This work offers a feasible solution for tunable WGM lasers and has significant application potential for preparing compact photonic elements.

## Data Availability

Data sharing not available.
